# Assessment of Vigilance Level during Work: Fitting a Hidden Markov Model to Heart Rate Variability

**DOI:** 10.3390/brainsci13040638

**Published:** 2023-04-07

**Authors:** Hanyu Wang, Dengkai Chen, Yuexin Huang, Yahan Zhang, Yidan Qiao, Jianghao Xiao, Ning Xie, Hao Fan

**Affiliations:** 1 Key Laboratory for Industrial Design and Ergonomics of Ministry of Industry and Information Technology, Northwestern Polytechnical University, Xi’an 710072, China; 2 Shaanxi Engineering Laboratory for Industrial Design, Northwestern Polytechnical University, Xi’an 710072, China; 3 Design Conceptualization and Communication, Faculty of Industrial Design Engineering, Delft University of Technology, 2628 CE Delft, The Netherlands; 4 Department of Ophthalmology, Shanghai General Hospital, Shanghai Jiao Tong University, Shanghai 200080, China; 5 Institute of Modern Industrial Design, Zhejiang University, Hangzhou 310007, China

**Keywords:** hidden Markov model, vigilance, heart rate variability, wearable device, psychomotor vigilance task, visual search task

## Abstract

This study aimed to enhance the real-time performance and accuracy of vigilance assessment by developing a hidden Markov model (HMM). Electrocardiogram (ECG) signals were collected and processed to remove noise and baseline drift. A group of 20 volunteers participated in the study. Their heart rate variability (HRV) was measured to train parameters of the modified hidden Markov model for a vigilance assessment. The data were collected to train the model using the Baum–Welch algorithm and to obtain the state transition probability matrix $\hat{A}$ and the observation probability matrix $\hat{B}$. Finally, the data of three volunteers with different transition patterns of mental state were selected randomly and the Viterbi algorithm was used to find the optimal state, which was compared with the actual state. The constructed vigilance assessment model had a high accuracy rate, and the accuracy rate of data prediction for these three volunteers exceeded 80%. Our approach can be used in wearable products to improve their vigilance level assessment functionality or in other fields that have key positions with high concentration requirements and monotonous repetitive work.

## 1. Introduction

Internet of Things (IoT) and sensor technologies can now monitor and evaluate an individual’s personal status while they are working [1]. However, an accessible product needs to be designed for people to easily know their measured current personal status data during work to understand workplace physiological conditions better.

Vigilance can be defined as the ability to achieve and maintain a state of high sensitivity to incoming stimuli. It is a measure of perceiving and responding to subtle changes that occur at random time intervals in a particular environment [2]. Vigilance is a special form of attention [3]. Continuous vigilance is related to types of work, such as aerospace, navigation, driving, etc. [4,5,6]. Caldwell found that official statistics indicate that low vigilance-caused fatigue is involved in at least 4–8% of aviation mishaps [7]. However, vigilance measurements are not directly available, which is taken by three main modalities: the subjective scale, experimental paradigm, and physiological signals [8].

Because vigilance levels are often linked to fatigue rate, some subjective scales for fatigue rate assessment are often used to assess vigilance. The most commonly used scales are: Karolinska sleepiness scale (KSS) and Stanford sleepiness scale (SSS). KSS uses nine levels to describe the degrees of fatigue: 1 = extremely awake, 5 = neither awake nor sleepy, and 9 = very drowsy (making a great effort to stay awake) [3]; furthermore, SSS uses seven levels to describe degrees of fatigue: 1 = fully awake, 3 = awake, 7 = close to sleep [9].

The experimental paradigm is used to evaluate vigilance levels when studying the impacts of sleep deprivation or human rhythm variation. The commonly used methods include the psychomotor vigilance task (PVT) and the Mackworth clock test (MCT). The PVT experiment was first used to assess decreased vigilance level because of sleep deprivation [10] and measure the speed of a participant’s psychomotor reactions [11]. Therefore, it depends on the participant’s responsiveness to stimuli. The main index is the response time of the participant, and the results are obtained through the repeated sampling of random stimuli [12]. The “gold standard” PVT-192 device accurately detects the vigilance of subjects over 10 min. Common indicators of PVT include the grand mean, and the PVT fastest RTs, slowest 10%, and lapse frequency [13]. Researchers attempted to migrate PVT to different hardware platforms, such as touch screens or PCs [14,15]. Compared with the PVT-192 device, the system can measure reaction times with an average delay of less than 10 ms; furthermore, the margin of error is comparable to the gold standard [14].

MCT is used to record participants’ response time and exact rates of response in small probability and target events, respectively, to evaluate the subjects’ current vigilance levels [16]. MCT is also involved in experimental psychology that explores the influence of long-term vigilance on signal detection [16].

Wearable devices constitute an emerging approach and are excellent candidates for supporting human activities and improving quality of life. They represent a new means of addressing the needs of many industries and have the potential to increase work efficiency among employees, improve workers’ physical well-being, and reduce work-related injuries [17]. Wearables are being used to provide various value-added services such as localization and navigation, financial payment, health monitoring, sport analytics, and medical insurance analytics [18]. Wearables are capable of continuously sensing, collecting, and uploading physiological data, improving quality of life in ways that smartphones alone cannot easily achieve. Currently, numerous models of wearable devices are capable of collecting physiological signals to assess vigilance, including ECG, EEG, and more.

At present, the relatively mature physiological parameters for vigilance detection mainly include ECG signals, EEG signals, and pulse waves. The electrocardiogram (ECG) changes with the successive excitation of the pacemaker, atrium, and ventricle during each heartbeat. The contraction of the heart produces a pulsation, which generates an electrical signal that can be conducted from the inside of the body to the body’s surface along special cardiomyocytes, where it can be detected by an ECG device. Yu et al. found that the R wave of the ECG signal can effectively distinguish the two states of sleep and wakefulness [19]. Zhao et al. measured drivers’ ECG and EEG during driving, and highlighted a correlation between the increase in driving duration and the decrease in a driver’s vigilance level and heart rate (HR) because the standard deviation of the RR interval tends to increase [20]. Al-Shargie et al. classified vigilance levels with an accuracy level exceeding eighty-nine percent by collecting EEG data and using relative wavelet transform entropy (RWTE), partial directional coherence (PDC), and graph theory analysis (GTA) [21]. They also proposed a method to improve the vigilance level through audio stimulation of pure tone at 250 Hz, which significantly reduced reaction time (RT) by forty-four percent and improved target detection accuracy by twenty-five percent [22]. Separating HRV (heart rate variability) from the ECG signal also produces a large amount of human physiological and psychological information. Many researchers argued that HRV signals can be effectively used to distinguish sleeping and waking states. Zhang observed that the main wave, heavy wave’s amplitude, and the conduction time of the pulse wave showed considerable differences between waking and sleeping states [23]. Some studies also adopted an eye-tracking method to detect vigilance levels. Bodala et al. defined a new measure called the ”fixation score” by the eye-tracking method and found that with decreases in vigilance, the fixation score also decreased [24].

The hidden Markov model (HMM) is a typical dynamic Bayesian network model, which is used to estimate the probability distribution of state transitions in the dynamic sequences of the measurement process and the probability of measurement output [25]. HMM is widely used in speech recognition, fault diagnosis, and other fields with high accuracy. HMM rests on the assumption that underlying processes produce time-changing observations with discrete hidden states. The measured process variable is regarded as the realization of the underlying stochastic process [26]. The difficulty lies in determining the hidden parameters of the process from the observable parameters and using these parameters for further analysis. HMM can reflect both the randomness and the potential structure of variables, showing a strong modelling ability for internal relations and random signals. Our study attempts to construct a modelling method to measure and evaluate the cognitive state by establishing a connection between the easily available HRV signal and a person’s implicit cognitive state—vigilance level. Therefore, HMM is a very effective biological modelling and computing tool.

Our study’s primary contribution is building HMM for vigilance assessments accomplished by extracting HRV information from human body ECG signals. This innovation improves the real-time performance of current vigilance measurement techniques, enabling the measurement of potential cognitive ability levels. Our method expands the cross-field knowledge domain of cognition assessment and computer science, providing new approaches for research and practical applications.

## 2. Related Works

Vigilance is the ability to sustain attention and remain alert to a particular stimulus over a prolonged period of time [27]. Numerous jobs in the fields of industry, the military, medicine, and education demand constant attention with varying levels of cognitive workload. Security personnel [28], workers in charge of watching security cameras or baggage screening experts, operating vehicles, working in real classroom settings [29], as well as industrial and air traffic control [30], are examples that require high levels of attention. Vigilance is necessary for these tasks to be completed with sufficient cognitive efficiency; however, assessing vigilance is a considerable problem.

In the past decade, machine learning methods have played important roles in the vigilance assessments [31,32,33]. Generally, these methods have five different phases to assess vigilance: (i) a sample acquisition focused on each task; (ii) signal pre-processing such as band-pass filtering; (iii) a feature extraction stage; (iv) a classification or regression step; and (v) a feedback phase [34]. For example, researchers proposed neural network methods for mental fatigue monitoring to assess vigilance, such as LRNN and BP [35]. However, these studies inadequately dealt with various vigilance assessment scenarios. Such neural network-based methods have an over-fitting problem due to the few-shot examples of each task. To address such a problem, the HMM method, which utilizes HRV parameters, can meet the need for continuous vigilance assessments.

According to the literature [36], performance in monotonous tasks is associated with an increase in the LF component of HRV. The low-frequency power spectrum of HRV reflects both sympathetic and parasympathetic activities, which jointly control the heart. This is the theoretical basis for our research. Therefore, our proposed method aims to provide vigilance level prediction, and may play a role in improving work performance and preventing staff from suffering accidents by combining wearable devices with prompt prewarning functions.

For general evaluation products already in application, relevant methods for monitoring mental fatigue focuses on stress; however, work performance is associated with several factors in addition to stress and, therefore, is more intuitive to vigilance level measurements [37]. With advances in ECG sampling technology, our method of obtaining HRV indicators by measuring ECG is also more convenient and easier to deploy in work scenarios than the traditional method.

The literature also used a combination of ECG and EMG signals to develop a system that could simultaneously detect low-level vigilance manifestations such as drowsiness and inattention [38]. The KNN method and linear and quadratic discriminant analysis were used to classify these features with a maximum accuracy of 96.75%. However, this method needs more sensors, and larger numbers and types of sensors pose a considerable challenge to wearable device design.

Existing studies on vigilance mainly distinguish between sleep and wakefulness and focus on the variation of signal characteristics in typical vigilance tasks, such as MCT. Few studies introduced new models to improve vigilance measurement accuracy. Therefore, the purpose of our study was to systematically examine the feasibility of using ECG signal and HMM for vigilance detection from the perspective of time- and frequency-domain characteristics, in addition to improving accuracy and broadening the application prospects of wearable products for vigilance monitoring.

## 3. Methods

**Figure 1:**
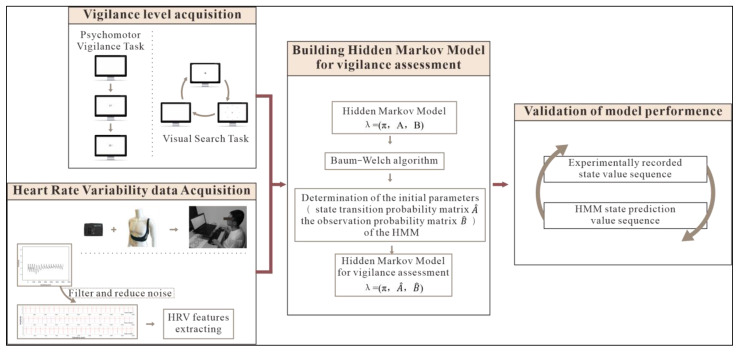
Research overview.

This section will introduce how volunteers’ HRV feature data were collected using wearable ECG signal sensors while conducting PVT and VST paradigms as the dataset. The dataset was used for training the HMM. Figure 1 presents an overview of the research in diagram form.

### 3.1. Assessment Protocol

In our study, we adopted the HMM because it describes the process of vigilance change as a process related to time sequence. On the other hand, HMM has the advantages of simple calculation and easy deployment. The assessment protocol will be detailed in the following sections.

To realize vigilance level classification, one needs to extract the signal features related to the state of vigilance. Our paper adopted the ECG as the object signal for extraction because it is closely associated with vigilance. The ECG measurement can effectively circumvent the EEG signal extraction process’s complexity and interference with participants.

#### 3.1.1. Psychomotor Vigilance Task

**Figure 2:**
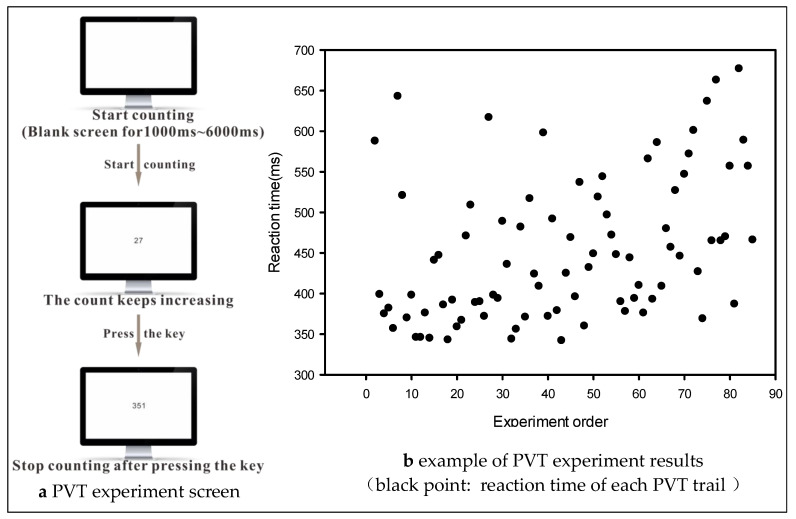
Psychomotor vigilance task experiment.

PVT is a visual response time measure that objectively quantifies human vigilance and fatigue by measuring visual response time to a simple and salient signal. We performed a 10 min standard PVT experiment to acquire data more conveniently. Figure 2 shows the PVT experiment. At the beginning of each trial, the screen appeared blank for 1000 to 6000 ms until a number of counts appeared. When the number appeared on the screen, the volunteer was asked to press the space bar as soon as possible. The number corresponded to the current time in milliseconds from the beginning of the digital presentation. The experiment automatically entered the next test trial if the volunteer pressed the space bar or gave no response at 5000 ms [13]. A PC based PVT version was used in our experiment. Using such a version was advantageous because of low hardware cost, high user familiarity, and the relative ease of software development [14].

#### 3.1.2. Visual Search Task

**Figure 3:**
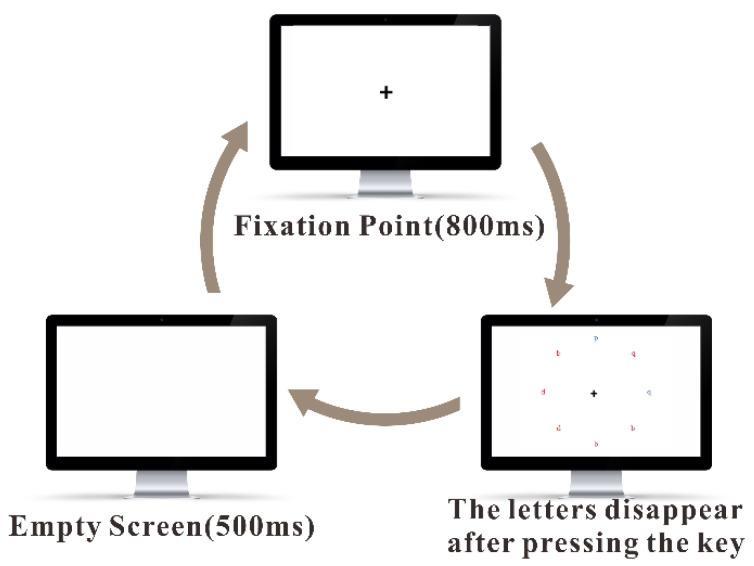
Visual search task experiment screen. When judging the color of the lowercase letter “p”, the volunteers were asked to press the “F” key if it was red; otherwise, press the “J” key.

We mainly used the visual search task (VST) to stimulate the volunteers’ cognitive load to artificially change their vigilance levels. Figure 3 first shows an 800 ms “+” fixation point, followed by a visual search stimulus, which was presented until the volunteers responded with a key press, upon which the search stimulus disappeared followed by the screen appearing blank for 500 ms. We instructed the volunteers to search for stimuli presented at the visual search interface using a guiding letter. When judging the color of the lowercase letter “p”, we asked the volunteers to press the “F” key if it was red; otherwise, we asked them to press the “J” key. The volunteers performed a 12-trial exercise session before the formal experiments, and the practice section similarly contained the stimuli for all conditions. The formal experiments started after the volunteers understood the experimental procedures and when the correct rate exceeded eighty percent. In our study, we mainly used VST to increase the variety of data changes [13]. The VST experimental results (such as reaction time and accuracy) did not affect our modelling. Our reason for adopting VST is that it was a structured experiment and easy to obtain; therefore, we could easily provide standard experimental materials to each participant.

#### 3.1.3. Participants

A total of 20 undergraduates participated in the experiment, including nine males and eleven females (aged 18–32, mean = 24, Standard Deviation = 2.60). None had a smoking history and reported normal hearing, vision, or corrected vision. The participants were required to maintain a regular sleep–wake period of at least one week before participating in the experiment and were not allowed to consume alcohol or functional beverages or participate in any high-intensity physical sports on the day of the experiment. The participants read and signed an informed consent form. They were also informed that they had the right to withdraw from the experiment anytime. We analyzed all reported data anonymously. Our study was approved by Northwestern Polytechnical University and complied with the Declaration of Helsinki.

#### 3.1.4. Instruments

We conducted our experiment in a quiet environment completely shielded from natural light using shade drapes. We completely replaced natural light with fixed artificial light sources. We used an EQ02 LifeMonitor ECG signal acquisition (EquivitalTM, Cambridge, UK). We used a well-established commercial finger-clip heart rate oximeter to simultaneously measure volunteers’ heart rates to compare with heart rates calculated from experimental data.

#### 3.1.5. Assessment Steps

**Figure 4:**
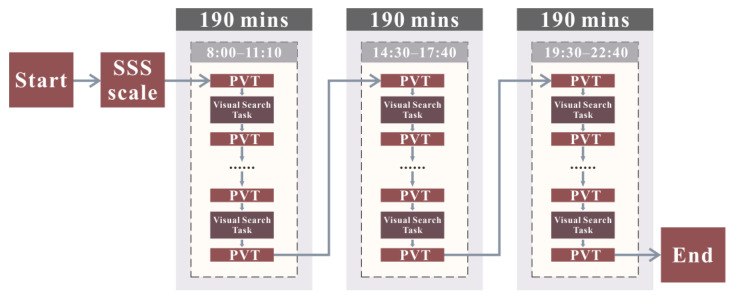
Flowchart of the assessment.

We artificially reduced volunteers’ vigilance levels using the visual search task to obtain vigilance data for different fatigue conditions. We asked the volunteers to attend the laboratory for data acquisition during three time periods: 8:00–11:10, 14:30–17:40, and 19:30–22:40. First, we tested the volunteers for subjective vigilance and drowsiness using the SSS. Next, they underwent a standard PVT trial for 10 min using a laptop computer. Immediately after that, they performed the visual search task; furthermore, once again, following the visual search task, vigilance was rated using PVT with a cycle of 10 trials ending at the simultaneous experiment’s conclusion. We recorded volunteer heart rates and HRV before combining them into physiological performance data [39]. From this, we obtained the experimentally recorded state sequence. Figure 4 shows the experimental flowchart with three segments of state sequence data. We completed the SSS, PVT, and VST experiments on the computer. Section 3.4 introduces specific data.

**Figure 5:**
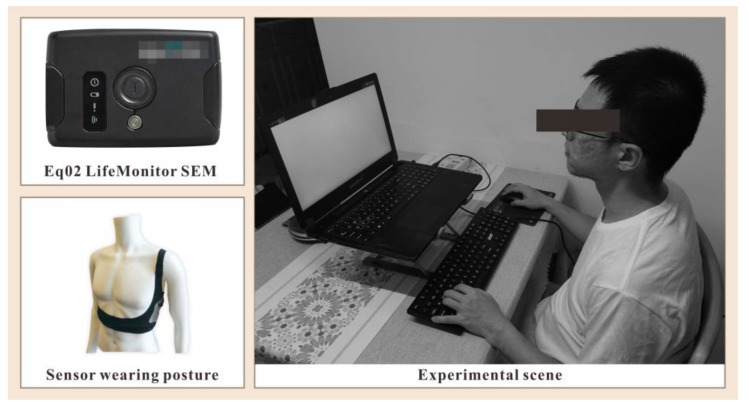
Experimental scene.

During the experiment, the volunteers kept their limbs still and their breaths even, and we kept them away from sources of electromagnetic interference, such as mobile phones, desk lamps, and electrical products. We transferred the collected ECG data to a computer through a cross-talk and saved it into table form to facilitate subsequent computerized processing. Figure 5 shows the acquisition scenes.

The ECG signal is characterized by low frequency and small amplitude, making it susceptible to external interference during data acquisition. Therefore, it is essential to preprocess the raw signal to eliminate noise pollution and baseline drift. Fortunately, ECG signal noise reduction methods have reached a high level of maturity. The empirical mode decomposition (EMD) method can eliminate high-frequency noise in the signal, while the wavelet denoising method can reduce power frequency interference. Applying wavelet multiresolution analysis (MRA) can reduce baseline drift, and the built-in accelerometer of the device can eliminate motion artifacts (MA), following its operational principle [40,41].

ECG data cannot be directly used in constructing an HMM; therefore, it is necessary to extract HRV features by means of dimensionality reduction. The key to extracting HRV features from the raw signal is to determine the main wave’s peak position in the R–R cycle. We utilized the differential threshold approach for this purpose. Next, we selected N cardiac cycles (i.e., the periodic time sequence of N heartbeats), each denoted as *t(n)*, *n* ∈ {1, *N*}, on the volunteers’ ECG waveforms. Then, we recorded the instantaneous HR(n) and mean heart rates *HR_MEAN*, as well as the difference between cardiac cycles, which we recorded as *δ*_(*n*)_ = *t*_(*n*)_ − *t*_(*n*−1)_. Standard deviation of the R-R (peak) interval (*SDNN*) and root mean square of the difference between adjacent R-R intervals (*RMSSD*) can be expressed in terms of the average cardiac cycle $\bar{\delta }$. We used the low-frequency power (*LFP*) indicator to calculate the integrated power in the low-frequency band of 0.04 Hz–0.15 Hz after performing a spectral transformation on the R–R interval sequence of the N cardiac cycles; furthermore, we used the high-frequency power (*HFP*) indicator to calculate the integrated power in the high-frequency band of 0.15 Hz–0.40 Hz. Table 1 shows the HRV features we used in our work.

### 3.2. Markov Chain Determination and Characteristic Parameter Processing

**Figure 6:**
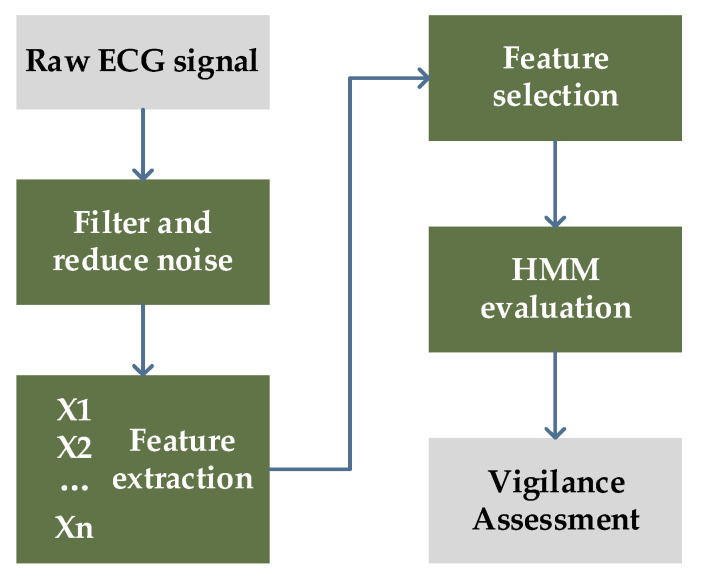
Modelling process of HMM for vigilance assessment.

The procedure for constructing the HMM for vigilance assessment was as follows: (1) we determined the initial model parameters λ = (*π*, *A*, *B*); (2) we used the Baum–Welch algorithm to train and obtain appropriate initial model parameters $\hat{\lambda }$ = ($\hat{\pi }$, $\hat{A}$, $\hat{B}$); (3) we used the Viterbi algorithm to input the observed value sequence into the established HMM for vigilance assessment to obtain the optimal state sequence, which was compared with the actual state sequence to estimate the model’s accuracy. Figure 6 shows the whole modelling process.

**Figure 7:**
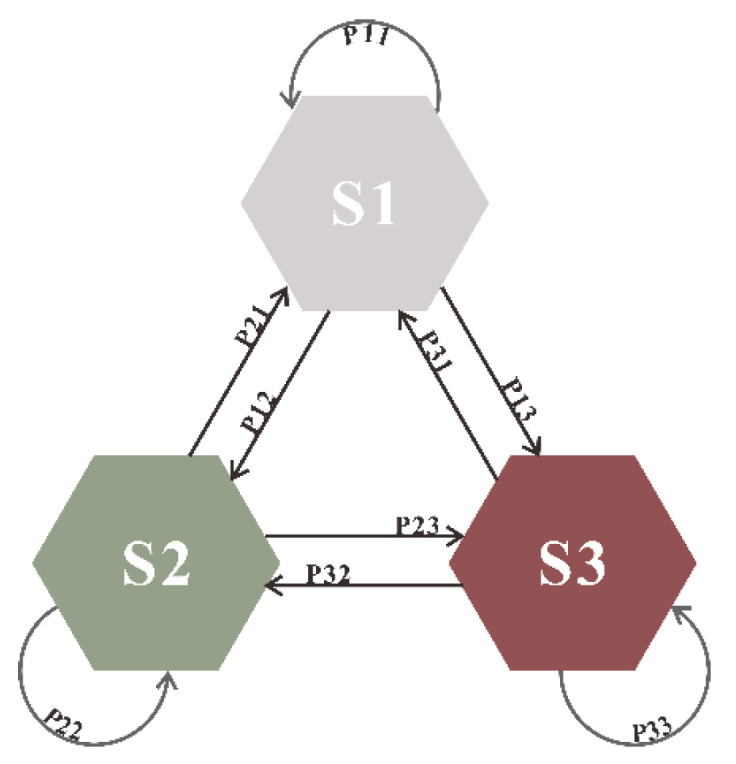
Schematic diagram of the Markov chain.

Because our study aimed to determine vigilance levels during working, we associated high, medium, and low vigilance levels with three hidden states (Figure 7). Considering the possibility that an individual may transition into a native or any other state from the current state at some point, the HMM allows for the transition of each state into the next or current state. State S1 denotes the state of low vigilance, S2 denotes the state of medium vigilance, and S3 denotes the state of high vigilance.

In our HMM approach to assess vigilance, we defined three hidden states corresponding to three observed variable states (1, 2, and 3). To determine the threshold of *SDNN* state segmentation, we used boxplots, which accurately and consistently depict the discrete data distribution. In analyzing the experimental data, we classified vigilance level states into three levels based on subjective scales and the fastest 10% of responses in the PVT, which reflected the participant’s level of sustained attention and were optimally represented by the activation of ongoing attention networks and the motor system cortex, as found in a previous study [27]. We then established the relationship between vigilance and *SDNN*, and Section 3.4 provides more details on the classification results.

### 3.3. Determination of the Initial Parameters of the HMM

The impact of the initial probability distribution of HMM on the final results of the model was contingent upon the nature of the data and the specific task. In certain scenarios, utilizing the average distribution as the initial probability distribution may not significantly affect the final model results, as the model is capable of learning more precise probability distributions through the training process [42]. Chen and Goodman demonstrated that utilizing the average distribution as the initial probability distribution did not have a detrimental impact on the modeling performance [43]. The reason for this is that the HMM model can improve its accuracy in probability distribution as the training progresses. Furthermore, Baum and Petrie noted that the initial probability distribution in HMM served merely as a starting point, as the model can dynamically adjust its parameters based on the data, rendering the impact of the initial probability distribution typically short-lived [44].

Since the initial values of entries in the initial state probability vector *π* and the state transition probability matrix *A* had little effect on the model training results [45], only the following conditions need to be met.

(1) $$\pi_{i}=P\left(q_{i}=S_{i}\right),\;1<i<N$$

(2) $$\sum_{i=1}^{N}\pi_{i}=1,\;0\leq \pi_{i}\leq 1$$

(3) $$A=\left[\begin{array}{ccc} a_{11} & \cdots & a_{1N}\\ \vdots & \ddots & \vdots \\ a_{N1} & \cdots & a_{NN} \end{array}\right]$$

(4) $$\sum_{i=1}^{N}a_{ij}=1,\;0\leq a_{ij}\leq 1$$

*π* and *A* can be considered randomly selected or uniformly taken. Because a left–right model is usually adopted in pattern recognition, we set the initial state probability vector *π_i_*, without making an estimate, to:

(5) $$\pi_{1}=1$$

(6) $$\pi_{i}=0,\;i=2,3,\ldots ,N$$

The values of entries in *A* were initialized by the principle of uniform distribution [46] by the following formula:

(7) $$a_{ij}=\frac{1}{\begin{array}{c} \mathrm{The}\;\mathrm{number}\;\mathrm{of}\;\mathrm{transfer}\\ \mathrm{paths}\;\mathrm{on}\;\mathrm{the}\;\mathrm{Markov}\;\mathrm{chain}\\ \mathrm{that}\;\mathrm{move}\;\mathrm{out}\;\mathrm{of}\;\mathrm{state}\;i \end{array}}$$

From the above analysis, there were three Markov chains, namely the high, medium, and low vigilance levels. Three transfer paths connect these states, so *a_ij_* = 1/3, i.e., the initial state transition probability matrix *A* is:

(8) $$A=\left[\begin{array}{ccc} a_{11} & \cdots & a_{1N}\\ \vdots & \ddots & \vdots \\ a_{N1} & \cdots & a_{NN} \end{array}\right]=\left[\begin{array}{ccc} 1/3 & 1/3 & 1/3\\ 1/3 & 1/3 & 1/3\\ 1/3 & 1/3 & 1/3 \end{array}\right]$$

Let S1, S2, and S3 represent the low, medium, and high vigilance levels, respectively. Each entry in A denotes the probability of transferring from a certain vigilance level to another. For example, *a*_13_ represents the probability of transferring from S1 to S3, whereas *a_31_* represents the probability of transferring from S3 to S1, and *a*_22_ represents the probability of transferring from S2 to S2.

We used experimental data to determine the initial entry values in the initial observation probability matrix B. We analyzed and extracted our acquired ECG signal and *SDNN* data, respectively, constructed a sample database for the HMM’s vigilance assessment, and calculated the values of individual entries *b*_ij_ in *B*, which denote the probability that the observed value is *j* when the state is *i*, by mathematical statistical means.

For example, to calculate *b*_11_ in *B*, select a sequence of observed values with a length of 30 as the statistical data sample:

O = [2, 3, 2, 2, 1, 2, 1, 2, 2, 2, 2, 3, 3, 1, 1, 2, 1, 1, 2, 2, 2, 1, 2, 3, 2, 3, 2, 3, 3, 1]

The state sequence of the corresponding experiment record at this time is:

Q = [2, 2, 1, 3, 2, 2, 3, 1, 2, 2, 2, 1, 1, 2, 1, 2, 2, 2, 3, 2, 3, 1, 3, 1, 2, 2, 2, 2, 2, 1]

*b*_11_ represents the probability that the observed variable is in state 1 when the human body is in the low vigilance level, i.e., the probability that the value in O is 1 when the value in Q is 1. In the above sequence of states, the number of 1 s is 8, the number of state 1 s and the observed value 1 s is 3, hence *b*_11_ = 3/8 = 0.38. We used the same method to determine the values of other entries in the observation probability matrix B.

We provided each volunteer with a sequence of observed values of length 30 and a state sequence upon completion of the trial. To avoid the influence of a single special datum’s training results on the model, we selected 10 random sets of data using the method described above for value initialization to obtain a sequence of observation of length 300, an experimentally recorded state sequence for modelling, and the initial observation probability matrix *B*. The Datasets section describes the specific result of *B*.

(9) $$B=\left[\begin{array}{ccc} b_{11} & b_{12} & b_{13}\\ b_{21} & b_{22} & b_{23}\\ b_{31} & b_{32} & b_{33} \end{array}\right]$$

We adopted the UMDHMM (hidden Markov model toolkit) lightweight C language version HMM package developed by Dr. Tapas Kanungo, the chief application scientist of Microsoft, to implement the algorithm. We optimized the values of entries in B during the modelling process. We describe the optimized results in the Datasets section.

### 3.4. Data Training

First, we determined the data length. The international standard duration of short-lasting data is generally 5 min and has many characteristics, such as easiness of grasp and control, and less susceptibility to external interference. It is widely used in many studies and clinical trials to analyze HRV data. In our study, the changes in vigilance were sensitive and susceptible to stimuli; therefore, we divided the data into multiple 1-min-long bins for processing and analysis by being intercepted to 6000 sampling sites in length.

**Figure 8:**
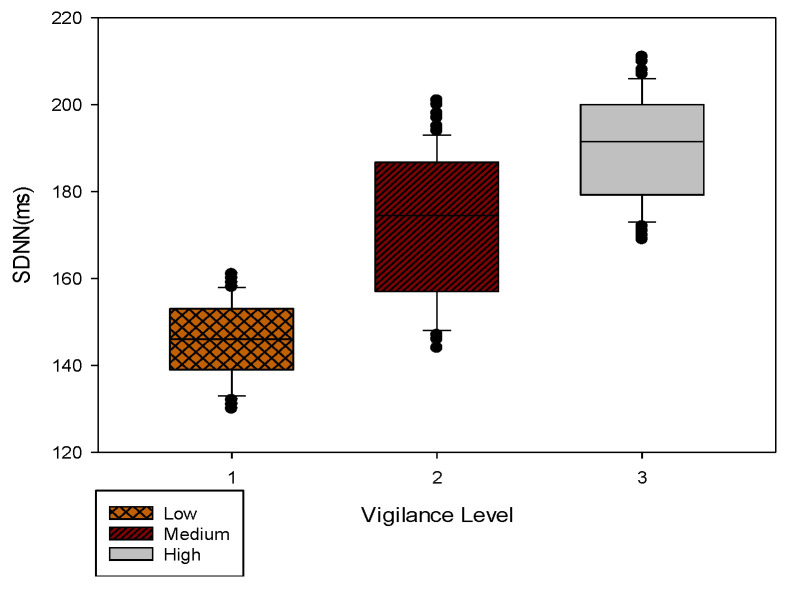
*SDNN* numerical statistics results.

Threshold segmentation was also needed for *SDNN*-vigilance levels. In general, the higher the HRV, the more active the vagus nerve and, hence, vigilance. We divided the sampled fastest 10% of responses into three phases, statistically analyzed the scale results, and set a threshold for the PVT results, at which the vigilance was higher and the PVT fastest 10% of responses was lower. The PVT fastest 10% of responses was divided into three levels, namely <400 ms, 400–500 ms, and >500 ms, based on questionnaire results, corresponding to high, moderate, and low vigilance levels, respectively. Figure 8 shows a boxplot of the *SDNN* numerical statistics results versus vigilance levels.

It can be seen from Figure 7 that the upper quartile of the low vigilance level was less than 153, whereas the lower quartile of the medium vigilance level was higher than 156, so the level segmentation threshold of *SDNN* was set to 155; the upper quartile of the medium vigilance level to 186, and the lower quartile of the high vigilance level to 179. Since this manuscript was committed to verifying whether the HMM could be used to predict the change in vigilance level, the state segmentation threshold of *SDNN* was set to 182. In future studies, more precise vigilance threshold segmentation methods will be investigated. The states of a low, medium, and high vigilance levels were set to S1, S2, and S3, respectively, for subsequent programming and postprocessing.

The matrix B in Formula (9) also needs to be calculated. The exact calculated result of *B* is:

$$B=\left[\begin{array}{ccc} b_{11} & b_{12} & b_{13}\\ b_{21} & b_{22} & b_{23}\\ b_{31} & b_{32} & b_{33} \end{array}\right]=\left[\begin{array}{ccc} 0.3662 & 0.4648 & 0.2690\\ 0.2436 & 0.5128 & 0.2436\\ 0.2877 & 0.4521 & 0.2603 \end{array}\right]$$

Finally, the Baum–Welch algorithm was employed to estimate ${\hat{a}}_{ij}$ and ${\hat{b}}_{j}\left(k\right)$ using a sequence of 300 observations, denoted as *O* = [*o*_1_, *o*_2_, …, *o*_T_], selected in the preceding section. This approach helped to obtain optimal solutions for the state transition probability matrix *A* and the observed probability matrix *B*, resulting in the values of $\hat{A}$ and $\hat{B}$.

$$\hat{A}=\left[\begin{array}{ccc} 0.0069 & 0.6255 & 0.3676\\ 0.0865 & 0.0077 & 0.9058\\ 0.3637 & 0.1571 & 0.4791 \end{array}\right]$$

$$\hat{B}=\left[\begin{array}{ccc} 0.3078 & 0.2082 & 0.4840\\ 0.6987 & 0.0157 & 0.2856\\ 0.0191 & 0.8510 & 0.1298 \end{array}\right]$$

Once the state transition probability matrix *A* and observation probability matrix *B* were optimized, the model could use them to identify the most probable sequence of states (i.e., levels of vigilance) based on a given sequence of observations (i.e., HRV data). In other words, by inputting a predetermined sequence of observations, the model can use the optimized matrices to determine the most likely corresponding sequence of states.

## 4. Results and Discussion

This section first verified the model’s accuracy, Hamming distance, and mean absolute error (MAE). By choosing three volunteers’ data, the data were verified and compared with SVM. Then, the evaluation metric in this paper was introduced. Finally, the limitation of our work is discussed.

Considering that the task’s setup conditions (e.g., stimulus duration and inter-stimulus interval) have the potential to influence the experimental results, we contrasted the results obtained by PVT and visual search task experiments with those obtained by the HMM as reference.

During the experiment, we found that the way mental states transform differed from individual to individual. A participant’s state of vigilance level within a single experiment will form 10 consecutive states based on the PVT experimental results. The participant will produce a state sequence with a length of 30 after completing the experiment on that day, such as the experimentally recorded state sequence Q = [3, 1, 2, 2, 2, 1, 2, 2, 2, 3, 1, 3, 2, 2, 2, 2, 3, 1, 2, 1, 1, 1, 3, 3, 2, 1, 1, 2], which shows the changes in vigilance level in the three experimental trials.

The model we trained was a non-individual model, meaning we did not process the data separately for each volunteer. Instead, we cut up all the data and used them collectively to build HMM, achieving an accuracy of 92.67% during the training process. We expected the model’s predictive accuracy to improve as we increased the number of subjects in our dataset. In the future, we can use this model directly for individual vigilance prediction without personal data.

**Figure 9:**
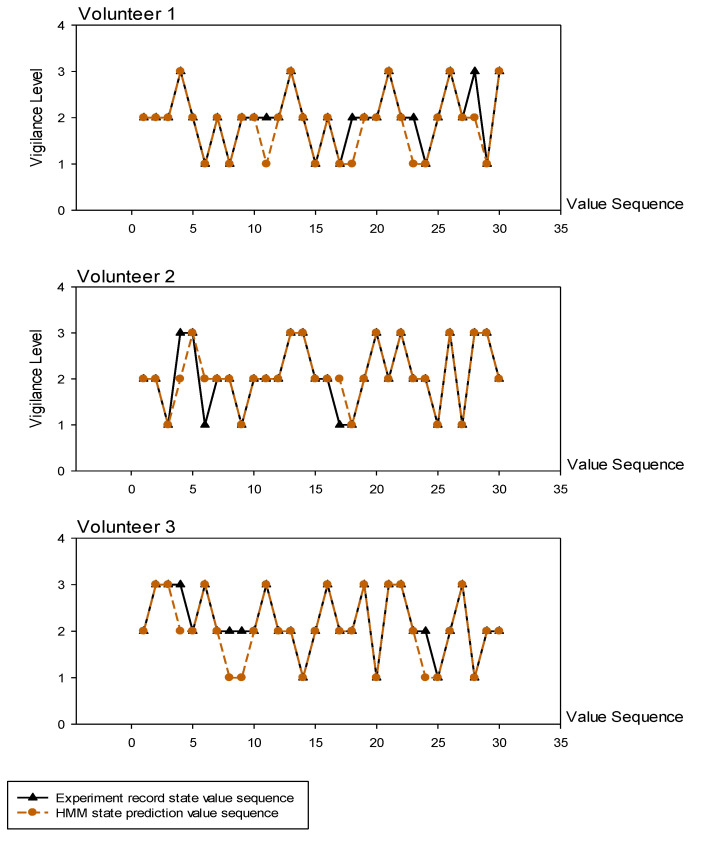
Validation results for vigilance assessment using hidden Markov model.

To evaluate the accuracy of our HMM approach in predicting vigilance, three volunteers who participated in the model evaluation through the PVT experiment were invited. The observed values of the volunteers in the PVT experiment were input into the HMM to obtain the prediction of vigilance. The results are shown in Table 2 and Figure 9. By comparing them to the state values in the PVT experiment, we obtained an average accuracy rate of 87.78%, demonstrating the efficacy of our method. Therefore, the vigilance assessment model based on the HMM that we constructed can very accurately detect changes in the human vigilance state without producing large deviations due to the different modes of human mental state transition.

Table 3 presents the model’s performance in predicting the vigilance level of three volunteers, as measured by the Hamming distance, accuracy, and mean absolute error (MAE). The MAE is a commonly used metric that quantifies the average magnitude of the errors between the predicted and true values of a model. On the other hand, the Hamming distance calculates the number of different characters in the same position between two strings of equal length.

In order to contrast the differences between the HMM and other algorithms, the SVM algorithm was used to make predictions according to the same section of experimentally recorded observation sequence. The support vector machine (SVM) is a popular machine learning algorithm used for classification and regression tasks. SVM can handle both linear and non-linear data. The key concept behind SVM is to maximize the margin between different classes by finding the optimal hyperplane that separates them. This hyperplane is chosen so that it is equidistant from the closest data points of each class, which are called support vectors. By maximizing the margin, SVM can achieve good generalization performance and avoid overfitting. The results of accuracy contrast are shown in Table 4, suggesting that the method adopted in our paper was superior to the SVM algorithm.

PVT and VST were adopted as our evaluation metrics. Our paper primarily focused on vigilance variation, and so, we chose the fastest 10% PVT reaction time as our model’s measure. Although there are various statistical techniques for PVT to assess the level of vigilance, we verified that the fastest 10% reaction time was essentially consistent with the participant’s subjective feelings of fatigue, which increased with the increases in task duration; however, this correlation was not affected by the task level [13].

VST was selected to modify volunteers’ vigilance level because there was little variation in the levels. According to our results, the vigilance level seemed to change irregularly; with respect to this phenomenon, we believe that the changes in vigilance level by the VST showed a form of periodicity. VST and other personal conditions affecting the volunteers may affect the vigilance level because PVT has no practice effect. We believe that personal conditions such as resting well or not before the experiment and other work loadings will influence vigilance levels in conjunction with VST. In future studies, we will try to analyze data to find periodicity.

Segmentation thresholds of vigilance levels were divided into wakefulness and fatigue (sleepiness) only in most current literature. However, during the experiment, we learned that different vigilance level states could still impact work performance, even in the waking state. Therefore, further studies are needed to determine more precise demarcation criteria.

PVT has considerable effects on volunteer behavior, and physiology, and it may cause subjective drowsiness and mental fatigue, as well as changes in autonomic function and the central nervous system. Our manuscript highlighted the use of physiological methods for vigilance measurements. Changes in vigilance levels were monitored based on indicators of autonomic nerve function, such as HRV. Changes in vigilance levels have a close relationship with physiological parameters, which serve as indicators of vigilance. The HMM is an effective vigilance level estimator.

The average identification accuracy rate and MAE for our HMM method was up to 87.78% and 0.12 in our experiment. MAE measures the average absolute error between the predicted values generated by a model and their actual values [47]. The results of the MAE indicated that the average difference between the predicted and actual values was relatively small. A smaller MAE value indicated a better prediction ability of the model. A minimum value of 0 indicated that the model’s predictions were completely accurate and free of error, whereas a larger value indicated that the model’s predictions deviated more from the actual values. However, the maximum value of MAE was not fixed and can vary depending on the range and variability of the data. Therefore, it is important to compare the MAE of different models using standardized metrics, such as relative error or mean percentage error, and take into account the context and characteristics of the data being analyzed. The results of Hamming distance suggest that the predicted vigilance state sequence is relatively close to the actual vigilance sequence. Our approach was compared with the SVM [48], and our results showed that the accuracy gaps between different algorithms were not apparent. However, HMM has higher accuracy than SVM. HMM can analyze the dynamic signals of time series, complete pattern recognition according to the relationships between adjacent states, and reflect the similarities between categories to a greater extent while ignoring differences between categories. By mapping the linearly inseparable samples in the low-dimensional space to the high-dimensional space, SVM separates similar samples with the largest possible Euclidean distance, which reflects the difference between categories to a greater extent. Both models have their advantages; furthermore, there are studies that combined the two algorithms to extract speech features or recognize driving intentions. Thus, in future research, the way that HMM and SVM cascade to further enhance the accuracy of vigilance assessment may be explored. Our study was aimed at workers in key positions of specific industries, such as the manned deep submersible or nuclear industries, which require high concentration levels when dealing with monotonous and repetitive work.

For the purpose of this paper, we were primarily interested in how wearable devices can be used for daily cognitive assessments based on collected physiological signals. Currently, the most commonly used civilian wearable devices are wristbands and smartwatches, e.g., the Empatica E4 Wristband contains an electrodermal activity (EDA) sensor that measures signals related to stress, engagement, and excitement [49]. However, it is worth noting that the hardware system of such sensors is complex, and as a research instrument, it does not provide support for further data availability. Alberdi et al. [50] published a review on stress recognition approaches where they gathered research on which signals were used and with which methods to recognize stress. The authors did not limit themselves to wearable devices to either measure or recognize inputs and stress, and so, the results with this paper do not directly match this paper, topic-wise. Nonetheless, this review still offers valuable insights into stress recognition approaches that could inform the development of wrist-wearable devices for stress recognition.

Based on our investigation, current wearable devices, particularly those designed for research purposes, tend to offer more diverse and accurate raw physiological data rather than sophisticated data analysis, but also provide a hardware foundation for our method. In contrast, wearable devices intended for daily use often focus on specific aspects of human activity, such as exercise or sleep tracking. Although there was considerable research on wearable devices, vigilance assessment was largely overlooked. Our research aimed to bridge this gap by developing algorithms embedded in hardware and exploring the potential for using wearable devices to assess cognitive abilities in real-world settings.

Our study’s limitations included the small sample size of 20 volunteers and the narrow range of vigilance levels. Finding statistically significant results was challenging due to the limited sample size, which restricted the experiment’s analytical capacity. Furthermore, while our paper aimed to comprehensively examine the availability of HMM for assessing vigilance, no further analysis of data changes during collection was performed. Additionally, a narrow focus was presented on predicting the mental state of only three individuals. As such, further research utilizing larger sample sizes and a more diverse range of participants is necessary to corroborate the findings and explore the potential applications of this approach.

In this paper, we adopted the method of average distribution for the initial probability distribution of HMM, which may have limited the accuracy of the model to a certain extent. In the subsequent study, we will discuss and improve the model’s accuracy by comparing the present model with models using other initial probability distribution methods.

The ECG collection device in our study was also only applicable in a lab environment, meaning that there is still a practical-perspective gap. Other devices and signals that are more convenient for real-world working environments will be tested in future work.

## 5. Conclusions

Long-term cognitive work induces a decrease in vigilance levels. In our paper, we used the HMM to improve the real-time performance and accuracy of current vigilance measurement techniques. We extracted the human body’s HRV information by experimentally collecting the body’s ECG signals while simultaneously recording volunteers’ mental states and building an HMM for vigilance assessments. We proved that HRV information may indicate vigilance levels, as well as the availability of the PVT in detecting vigilance. Our experimental vigilance assessment model had a high accuracy rate that exceeded eighty percent. The method we adopted also had a higher accuracy level compared with the SVM. Our experimental results showed that our constructed vigilance assessment model had high accuracy rates. Our method provided new techniques for measuring potential cognitive ability levels and expanded the cross-field knowledge domain of cognition assessment and computer science. Our approach can be used in wearable products to improve their vigilance level assessment functionality. In particular, our research has broad application prospects in the navigation, aerospace, and nuclear industries, as well as other fields that have key positions with high concentration requirements and monotonous repetitive work.

## Figures and Tables

**Table 1:** Heart Rate Features (*HR*(*n*): heart rate; *HR_MEAN*: Mean heart rate; *SDNN*: Standard deviation of the R-R interval; *RMSSD*: Root mean square of the difference between adjacent R-R intervals; *LFP*: Low-frequency power; *HFP*: High-frequency power).

Feature	Feature Type	Meaning	Calculation
*HR(n)*	Heart rate indicator	Instantaneous heart rate	$HR(n)=\frac{60}{t_{\left(n\right)}}$
*HR_MEAN*	Heart rate indicator	Mean heart rate	${HR\_MEAN=\bar{HR}}_{\left(n\right)}$
*SDNN*	Heart rate variability indicator	Standard deviation of the R-R (peak) interval	$SDNN=\sqrt{\frac{1}{N-1}\sum_{n=2}^{N}{\left(\delta_{\left(n\right)}-\bar{\delta }\right)}^{2}}$
*RMSSD*	Heart rate variability indicator	Root mean square of the difference between adjacent R-R intervals	$RMSSD=\sqrt{\frac{1}{N-2}\sum_{n=3}^{N}{\left(\delta_{\left(n\right)}-\delta_{\left(n-1\right)}\right)}^{2}}$
*LFP*	Heart rate variability indicator	Low-frequency (0.04 Hz–0.15 Hz) power	$LFP=\sum P_{\left(W\right)},\;0.04<W<0.15$
*HFP*	Heart rate variability indicator	High-frequency (0.15 Hz–0.4 Hz) power	$HFP=\sum P_{\left(W\right)},\;0.15<W<0.40$
*LFP/HFP*	Heart rate variability indicator	/	/

As vigilance declined, *SDNN*, *LFP*, and *LFP/HFP* considerably increased, *HFP* also noticeably decreased, and all showed significant linearities. Hence, we chose *SDNN* from among the above indicators, which we used as an observation matrix parameter in the HMM.

**Table 2:** Validation results for vigilance assessment using hidden Markov model.

Experimentally Recorded Observation Sequence										Experimentally Recorded State Value Sequence										HMM State Prediction Value Sequence										Accuracy
2	2	2	2	3	2	2	1	2	2	2	2	2	3	2	1	2	1	2	2	2	2	2	3	2	1	2	1	2	2	86.67%
2	1	3	3	3	2	2	2	3	2	2	2	3	2	1	2	1	2	2	2	1	2	3	2	1	2	1	1	2	2	
3	2	3	2	3	2	2	3	2	1	3	2	2	1	2	3	2	3	1	3	3	2	1	1	2	3	2	2	1	3	
1	2	1	1	3	2	2	2	1	2	2	2	1	3	3	1	2	2	1	2	2	2	1	2	3	2	2	2	1	2	90.00%
2	3	1	2	2	3	2	3	1	2	2	2	3	3	2	2	1	1	2	3	2	2	3	3	2	2	2	1	2	3	
3	3	3	1	2	2	1	2	3	1	2	3	2	2	1	3	1	3	3	2	2	3	2	2	1	3	1	3	3	2	
2	2	3	1	2	1	2	2	2	2	2	3	3	3	2	3	2	2	2	2	2	3	3	2	2	3	2	1	1	2	86.67%
2	1	2	2	1	2	3	2	1	2	3	2	2	1	2	3	2	2	3	1	3	2	2	1	2	3	2	2	3	1	
1	2	3	1	2	1	2	1	2	1	3	3	2	2	1	2	3	1	2	2	3	3	2	1	1	2	3	1	2	2	

**Table 3:** Results of the testing hamming distance, accuracy, and mean absolute error MAE of hidden Markov model.

	Hamming Distance	Accuracy	MAE
Volunteer 1	4	86.67%	0.1333
Volunteer 2	3	90.00%	0.1000
Volunteer 3	4	86.67%	0.1333

**Table 4:** Results of contrast between hidden Markov model and support vector machine.

	HMM										SVM									
Experimental record observation sequence	2	2	2	2	3	2	2	1	2	2	2	2	2	2	3	2	2	1	2	2
	2	1	3	3	3	2	2	2	3	2	2	1	3	3	3	2	2	2	3	2
	3	2	3	2	3	2	2	3	2	1	3	2	3	2	3	2	2	3	2	1
Experiment record state value sequence	2	2	2	3	2	1	2	1	2	2	2	2	2	3	2	1	2	1	2	2
	2	2	3	2	1	2	1	2	2	2	2	2	3	2	1	2	1	2	2	2
	3	2	2	1	2	3	2	3	1	3	3	2	2	1	2	3	2	3	1	3
prediction value sequence	2	2	2	3	2	1	2	1	2	2	2	2	2	2	2	1	2	1	2	2
	1	2	3	2	1	2	1	1	2	2	1	2	3	2	1	2	1	1	2	2
	3	2	1	1	2	3	2	2	1	3	3	2	1	1	2	3	2	2	1	3
Accuracy	86.67%										83.33%									

## Data Availability

Not applicable.
